# Risk factors of perioperative complications for posterior spinal fusion in degenerative scoliosis patients: a retrospective study

**DOI:** 10.1186/s12891-018-2148-x

**Published:** 2018-07-19

**Authors:** Hai Wang, Zheping Zhang, Guixing Qiu, Jianguo Zhang, Jianxiong Shen

**Affiliations:** 1 Department of Orthopaedic Surgery, Peking Union Medical College Hospital, Peking Union Medical College and Chinese Academy of Medical Sciences, No. 1 Shuaifuyuan, Beijing, 100730 China; 2 Department of Orthopaedic Surgery, Beijing Puren Hospital, Beijing, China

**Keywords:** Degenerative scoliosis, Perioperative complication, Risk factors, American standards association (ASA) classification, Operation duration (OD)

## Abstract

**Backgrounds:**

Rare study has been conducted to detect risk factors of perioperative complications, which are closely related to preoperative status of the patients and surgical stress. The aim of this study is to detect these relationships in degenerative scoliosis (DS) patients.

**Methods:**

Perioperative complications of 226 cases with DS (56 males and 170 females; 65.5 ± 8.1 years old), who accepted posterior fusion in our hospital from January, 2013 to July, 2017, were retrospectively reviewed. Potential risk factors were first compared between patients with or without perioperative complications using student t test or Chi-squared test. Then, the unevenly distributed variables between the two groups were analyzed with binary logistic regression model.

**Results:**

All patients separately underwent decompression with short limited instrumentation (116, 51.3%) or with long instrumentation for correction (110, 48.7%). The mean operation duration (OD) was 216.9 ± 64.2 min and the average amount of bleeding was 587.4 ± 357.2 ml. 44 cases (19.5%)suffered from the complications during the perioperative phase, including incision complications (5.3%), urinary infection (3.5%), dura tears/cerebrospinal fluid (CSF) leakages (3.5%) and new neurological deficits (2.7%). Hospital stay was significantly extended for the complications (*p* < 0.001). Univariate analysis showed that OD (*p* < 0.001), bleeding (*p* = 0.014), American Standards Association (ASA) grade > 2 (*p* = 0.011) and RBC transfusion≥4 U(*p* = 0.028) were associated with these complications. Multivariate logistic regressions revealed that only ASA grade > 2(*p* = 0.011, Odds Ratio[OR] = 4.104, 95% Confidence Interval[CI] = 1.413~ 11.917) and OD (*p* = 0.013, OR = 2.697, 95% CI = 1.233~ 5.899) were the independent risk factors.

**Conclusions:**

The high morbidity of perioperative complications for posterior spinal fusion would significantly extend hospital stay of DS patients. It was independently related to higher ASA grade and longer OD.

## Background

Degenerative scoliosis (DS), firstly described as “de novo” scoliosis, is a scoliosis caused by progressive degeneration of the spine in individuals without preexisting spinal deformity [1]. Most patients suffered from severe low back pain, nerve root compression symptoms and/or neurogenic claudication. Surgery is the most common therapy for patients who have failed conservative treatment, which can significantly alleviate the pain and improve the quality of life [2]. With the population aging, there is an associated rise in the prevalence of degenerative spinal disorders, including DS [3]. The demand for surgical treatment for DS had increased, with an increase of 3.4 times in more than 60 years of age from 2004 to 2011 according to the Scoliosis Research Society (SRS) database [4].

Surgical management of DS in the elderly population represents a challenge for spinal surgeons in clinic with the postoperative complication rate of 49% [2]. Perioperative complications accounted for more than half of postoperative complications [5], and were closely related to preoperative status of the patients and surgical stress [6]. Understanding risk factors of perioperative complications could help to optimize treatment plan and improve cost-effectiveness. Up to now, there are few reports about the relationship between the perioperative complications and their risk factors.

The aim of this study is to report and analyze the perioperative complications and the potential risk factors related to preoperative status of the patients and surgical stress in DS patients.

## Methods

### Patients

A total of 226 consecutive patients with DS, who underwent posterior spinal fusion in our medical center from January, 2013 to July, 2017, were retrospectively reviewed. The patients who received minimally invasive surgery or with malignant tumor were excluded. General Characteristics, including age, sex, body mass index (BMI), smoking history, medication history (especially for insulin), comorbidities, lumbar operation history, preoperative hemoglobin (Hgb) and hospital stay, were charted. American Society of Anesthesiologists (ASA) grade was evaluated by two anesthetists.

### Surgical procedures

X-rays, computerized tomography, and magnetic resonance imaging were routinely performed before operation. The surgical method was decided by all spine experts in our department based on its symptoms, signs, and images according to Lenke-Silva’s suggestions in combination with its age, medical comorbidities, and socioeconomic status [7, 8]. Surgical methods included decompression with limited short fusion and decompression with long fusion for correction. Fusion levels, iliac fixation, operation duration (OD), bleeding, and homologous red blood cells (RBC) transfusion were recorded after surgery. All perioperative complications occurring within 30 days after the operation were also collected in detail.

### Statistic analysis

Means and proportions were used to describe the overall sample as well as the groups identified by the presence (Group A) or absence (Group B) of perioperative complications. The quantitative variates (age, BMI, preoperative Hgb, OD, bleeding, and hospital stay) were compared between the two groups (Groups A&B) using student t test. The qualitative variates (sex [male or female], smoking [yes or no], ASA grade [1~ 2 or > 2], medical comorbidities [with or without heart disease/ respiratory disease/ diabetes mellitus(DM)/ insulin-dependent DM/immune disease], allograft [yes or no], RBC transfusion [< 4 U or ≥ 4 U], surgical method [short fusion (≤3 levels) or long fusion (> 3 levels) with correction], interbody fusion [yes or no], iliac fixation [yes or no], osteotomy [yes or no] and lumbar operation history [yes or no]) were compared by using Chi-square tests (Fisher exact tests where appropriate). All unevenly distributed variates between these two groups (Groups A&B) were analyzed with binary logistic regression model. The selection method was ENTER. Statistical analysis was performed with SPSS version 19.0 software (IBM Company, Armonk, New York, USA). All the potential risk factors were also analyzed between the patients with one complication and with multiple complications. *P* < 0.05 was set as significant difference.

## Results

### General characteristics

A total of 226 patients with DS were enrolled in this study, including 56 males and 170 females. Their mean age was 65.5 ± 8.1 years old. The average hospital stay was 15.9 ± 7.6 days. There were 133 cases (58.8%) with medical comorbidities, including 116 cases (51.3%) with heart disease, 35 cases (15.5%) with DM (8 cases with insulin-dependent DM), 11 cases (4.9%) with respiratory disease, and 11 cases (4.7%) with immune disease. Among them, there were about 34 cases (15.0%) with more than two medical morbidities. 7 cases (3.1%) had the history of lumbar surgery.

### Surgical characteristics

Decompression with limited short fusion was done in 116cases (51.3%), and decompression with long fusion and correction of deformity was done in 110 cases (48.7%). Osteotomy was performed in 7 cases (3.1%), including 4 Smith-Peterson osteotomies and 3 pedicle subtraction osteotomies. The mean OD was 216.9 ± 64.2 min and the average amount of bleeding was 587.4 ± 357.2 ml. 142 cases (62.8%) used autologous blood transfusion system and 79 cases (35.0% accepted allogeneic blood transfusion with RBC of 2.8 ± 1.3 U and plasma of 374.5 ± 122.4 ml.

### Perioperative complications

Perioperative complications occurred in 44 patients (19.5%) within 30 days after operation, including 12 cases (5.3%) with incision complications, 8 cases (3.5%) with urinary infections, 8 cases (3.5%) with dura tears/cerebrospinal fluid (CSF) leakages, 6 cases (2.7%) with new neurological deficits and others (Table 1). The incision complications included 7 superficial infections/fat liquefactions, 4 deep infections and 1 hematoma. 5 patients (2.2%) suffered from more than 2 complications, including 1 case with superficial infection and urinary infection; 1 case with deep infection, urinary infection, CSF leakage, and central nervous system infection; 1 case with deep infection and sepsis; 1case with superficial infection, pleural effusion,atelectasis and pulmonary embolism and 1case with pleural effusion and pulmonary infection. 3 patients (1.3%) underwent unplanned second surgeries for their complications, including 2 deep infections and 1new neurological deficit because of poor screw position. One patient with incision hematoma accepted the interventional puncture therapy.

**Table 1:** Perioperative complications of degenerative scoliosis within postoperative 30 days

Complications	Patients (n)
Incision complications	12 cases
Superficial infection/ fat liquefaction	7 cases
Deep infection	4 cases
Hematoma	1 case
Urinary infection	8 cases
Dura tear/cerebrospinal fluid leakage	8 cases
New neurological deficits	6 cases
Respiratory complications	4 cases
Pulmonary infection	3 cases
Pleural effusion	2 cases
Atelectasis	1 cases
Myocardial ischemia	2 cases
Venous thromboembolism	3 cases
Plexus venosus leg muscle thrombosis	2 cases
Pulmonary embolism	1 cases
Sepsis	2 cases
Delirium	1 cases
Abnormal glutamic-pyruvic transaminase	2 cases
Central nervous system infection	1 case
Cerebral infarction	1 case
Total	44 cases

### Statistic analysis

Hospital stay of the patients was significant prolonged by their perioperative complications (14.3 ± 5.3 vs 22.2 ± 11.6 days, *p* < 0.001). As shown in Table 2, the distributions of bleeding (*p* = 0.014), OD (*p* < 0.001), ASA grade (*p* = 0.011), and RBC transfusion (*p* = 0.028) were significantly different between the patients with and without perioperative complications. In order to eliminate influences of farraginous factors, a binary logistic regression model was used for multivariate analysis of unevenly distributed variates. The results showed that ASA grade > 2 (*p* = 0.009, Odds Ratio[OR] = 4.104, 95% Confidence Interval[CI] =1.413~ 11.917) and OD (*p* = 0.013, OR = 2.697, 95% CI = 1.233~ 5.899) were two independent risk factors (Table 3). Further, the risk factor of multiple complications was also evaluated. But there was no significant one to be associated with multiple complications (Table 4).

**Table 2:** The results of Univariate analysis between the patients with or without perioperative complications

Variates	Perioperative complications		*P* value
	No (*n* = 182)	Yes (*n* = 44)	
Age(years)	65.6 ± 8.2	65.1 ± 7.9	0.729
Preoperative Hgb(g/L)	134.6 ± 13.9	131. 0 ± 14.2	0.130
BMI(kg/m^2^)	25.6 ± 3.7	26.8 ± 3.6	0.065
Bleeding(ml)	558.8 ± 339.7	705.7 ± 405.1	0.014
OD(minutes)	208.6 ± 58.1	251.7 ± 77.2	0.000
Hospital stay(days)	14.3 ± 5.3	22.2 ± 11.6	0.000
Sex			
Female	135(79.4%)	35(20.6%)	0.459
Male	47(83.9%)	9(16.1%)	
ASA grade			
1~ 2	172(82.7%)	36(17.3%)	0.011*
> 2	10(55.6%)	8(44.4%)	
Heart disease			
No	91(82.7%)	19(17.3%)	0.417
Yes	91(78.4%)	25(21.6%)	
Respiratory disease			
No	173(80.5%)	42(19.5%)	1.000*
Yes	9(81.8%)	2(18.2%)	
DM			
No	151(79.1%)	40(20.9%)	0.191
Yes	31(88.6%)	4(11.4%)	
Insulin-dependent DM			
No	176(80.7%)	42(19.3%)	0.655*
Yes	6(75.0%)	2(25.0%)	
Immune disease			
No	174(80.9%)	41(19.1%)	0.451*
Yes	8(72.7%)	3(27.3%)	
Smoking			
No	161(82.1%)	35 (17.9%)	0.118
Yes	21(70.0%)	9(30.0%)	
Allograft			
No	54(79.4%)	14(20.6%)	0.780
Yes	128(81.0%)	30(19.0%)	
RBC transfusion			
< 4 U	167(82.7%)	35(17.3%)	0.028*
≥ 4 U	15(62.5%)	9(37.5%)	
Surgical method			
Short fusion	96(82.8%)	20(17.2%)	0.385
Long fusion	86(78.2%)	24(21.8%)	
Interbody fusion			
No	166(81.8%)	37(18.2%)	0.170*
Yes	16(69.6%)	7(30.4%)	
Osteotomy			
No	176(80.4%)	43(19.6%)	1.000*
Yes	6(85.7%)	1(14.3%)	
Lumbar operation history			
No	175(80.3%)	43(19.7%)	1.000*
Yes	7(87.5%)	1(12.5%)	
Iliac fixation			
No	177(80.8%)	42(19.2%)	0.624*
Yes	5(71.4%)	2(28.6%)	

*Fisher exact test

**Table 3:** The results of binary logistic regression analysis of unevenly distributed variates between the patients with or without perioperative complications. (method = “Enter”)

Variates	*P* value	Odds Ratio (95% Confidence Interval)
Bleeding	0.694	1.000(0.999~ 1.001)
RBC transfusion	0.274	1.840(0.616~ 5.494)
ASA grade	0.009	4.104(1.413~ 11.917)
OD	0.013	2.697(1.233~ 5.899)

**Table 4:** The results of Univariate analysis between the patients with one or multiple perioperative complications

Variates	Perioperative complications		*P* value
	< 2 (*n* = 39)	≥ 2 (*n* = 5)	
Age(years)	65.3 ± 7.9	64.0 ± 9.6	0.738
Preoperative Hgb(g/L)	130.7 ± 14.5	133.4 ± 13.3	0.694
BMI(kg/m^2^)	26.7 ± 3.6	27.6 ± 3.8	0.586
Bleeding(ml)	718.0 ± 395.8	610.0 ± 512.8	0.581
OD(minutes)	250.3 ± 69.8	263.0 ± 132.2	0.732
Sex			
Female	31(88.6%)	4(11.4%)	1.000*
Male	8(88.9%)	1(11.1%)	
ASA grade			
1~ 2	32(88.9%)	4(11.1%)	1.000*
> 2	7(87.5%)	1(12.5%)	
Heart disease			
No	17(89.5%)	2(10.5%)	1.000*
Yes	22(88.0%)	3(12.0%)	
Respiratory disease			
No	37(88.1%)	5(11.9%)	1.000*
Yes	2(100.0%)	0(0.0%)	
DM			
No	36(75.0%)	1(10.0%)	0.394
Yes	3(88.6%)	4(25.0%)	
Insulin-dependent DM			
No	37(88.1%)	5(11.9%)	1.000*
Yes	2(100.0%)	0(0.0%)	
Immune disease			
No	36(87.8%)	5(12.2%)	1.000*
Yes	3(100.0%)	0(0.0%)	
Smoking			
No	30(85.7%)	5(14.3%)	0.566*
Yes	9(100.0%)	0(0.0%)	
Allograft			
No	12(85.7%)	2(14.3%)	0.647*
Yes	27(90.0%)	3(10.0%)	
RBC transfusion			
< 4 U	31(88.6%)	4(11.4%)	1.000*
≥ 4 U	8(88.9%)	1(11.1%)	
Surgical method			
Short fusion	18(90.0%)	2 (10.0%)	1.000*
Long fusion	21(87.5%)	3(12.5%)	
Interbody fusion			
No	32(86.5%)	5(13.5%)	0.574*
Yes	7(100.0%)	0(0.0%)	
Osteotomy			
No	38(88.4%)	5(11.6%)	1.000*
Yes	1(100.0%)	0(0.0%)	
Lumbar operation history			
No	38(88.4%)	5(11.6%)	1.000*
Yes	1(100.0%)	0(0.0%)	
Iliac fixation			
No	38(90.5%)	4(9.5%)	0.217*
Yes	1(50.0%)	1(50.0%)	

*Fisher exact test

## Discussion

There was a relative higher morbidity of perioperative complications for DS, similar to that of postoperative complications. The morbidities of perioperative complications were about 20.4% in this study and 29.8% in Cho’s report [5]. They would significantly prolong the hospital stay (*p* < 0.001), and consequently increase the medical cost, and lower the postoperative satisfaction. Thence, it was a major challenge for spinal surgeons to deal with perioperative complications in DS patients.

Incision complications were the most common perioperative complications in DS patients (5.3%). Although some patients lack bacteriological evidence of infection after superficial fat liquefaction, we still considered that there was a high possibility of superficial infections. Therefore, superficial fat liquefaction and infections were put together, accounting for 58.3% of the total incision complications. Most of them could be healed by dressing and antibiotic therapy. The key points were close observation, early detection and treatment to prevent spreading to the deep structure. For deep infections, vacuum-assisted closure device was recommended, because it could significantly reduce the rate of reoperation of deep infection after spinal internal fixation, shorten the use of antibiotics, improve the retention rate of internal fixation [9]. In this study, 4 cases with deep infection were cured by the combination of vacuum-assisted closure device and systemic antibiotic therapy.

Urinary infection was another common complication of DS with the incidence of 3.5% in this study. It was one of the risk factors for incision infection after posterior spinal fusion [10]. There were 2 cases with incision infections following urinary infections in this study. Therefore, it was very important to prevent and early detect urinary infection. Most nosocomial urinary infections were associated with indwelling urinary catheters, and its incidence could be significantly reduced by shortening indwelling time [11]. It was suggested to remove urinary catheter as soon as possible.

Dural tear /CSF leak was one of the major complications of DS. The incidence was 3.5%, similar to 2.9% for adult scoliosis according to the SRS database [12]. It might lead to incision complications, pseudomeningocele, cerebrospinal fluid sinuses, arachnoiditis, meningitis, epidural abscess and psychiatric symptoms [13]. Once dural tear was found during operation, it was suggested to intraoperatively repair as far as possible [14]. The other recommended measures included suturing the para-spinal muscle to reduce the dead space, suturing the incision closely, and lying supine for 4–7 days after operation to reduce hydrostatic pressure.

New neurological deficits were common complications of concern in DS patients. The morbidity was 2.7% in this study, in accordance with 2.0% reported by SRS [12]. They were mostly associated with the performance of these extensive decompressions because DS patients often had significant spinal canal stenosis [15]. Most of them were transient nerve root complications and only needed conservative treatment [16]. But it was still necessary to be careful about the position of implants. There was one new neurological deficit for poor position of screw in this study. For these cases, it was very important to detect and address them to keep more neurological functions as early as possible.

Perioperative complications were associated with preoperative status of the patients and surgical stress [6]. Some variates about preoperative status and surgical stress were considered as potential risk factors of perioperative complications in this study, referring to the previous related studies [6, 17, 18]. Univariate analysis results showed that bleeding (*p* = 0.014), OD (*p* < 0.001), ASA grade (*p* = 0.011), and RBC transfusion (*p* = 0.028) unevenly distributed between patients with and without perioperative complications. Further, multivariate regression analysis results revealed that only ASA grade > 2 (*p* = 0.009) and OD (*p* = 0.013) were the independent risk factors.

The ASA classification system was first proposed in 1963, on behalf of the patient’s preoperative physical status [19]. Compared to other grading system (such as Acute Physiology and Chronic Health Evaluation II score, Physiological and Operative Severity Score for the Enumeration of Mortality and Morbidity, et al.), it was simple and easy to obtain [20]. The ASA grade had been confirmed to be associated with the morbidity and mortality in spinal surgery before [21, 22]. Its close relationship with the occurrence of postoperative complications in DS had also been reported [17]. In this study, the higher ASA grade (*p* = 0.009) was further founded to be related to the morbidity of perioperative complications in DS. It would be helpful in counseling individuals on risks of morbidity for perioperative complications.

Operative duration (*p* = 0.013) was the other independent risk factor for the development of perioperative complications in DS patients. First, it would likely result in higher incision infection rates. Because the surgical site exposed longer to the operating room surroundings with OD increasing. There was a significant correlation between the length of time that the sterile tray is exposed to the air and the contamination rate of the instruments [23]. Second, the duration of bleeding in the wound site would lead excessive intraoperative blood loss, which increased allogeneic blood transfusion. That’s why bleeding (*p* = 0.694) and RBC transfusion (*p* = 0.274) were confounding factors of perioperative complications in this study. Third, it was also associated with overall complications, medical complications, and surgical complications as reported before [24]. Furthermore, OD was prone to be extended by the complexity of the type of procedure or patient health, level of training and expertise, and multilevel fusions [25].

As the results revealed that ASA > 2 and long OD were two independently risk factors of perioperative complications in this study. Thence, the surgical strategies should be optimized to shorten OD for those patients with ASA > 2 after preoperative evaluation, such as decompression without fusion or with short fusion, or minimally invasive surgery. In this study, the surgical method had been optimized according to its age, medical comorbidities, and socioeconomic status based on Lenke-Silva’s suggestions. For some high-risk patients, short fusion was selected. That might be why long fusion was not related to perioperative complications.

There were several limitations for this study. The major limitation was the small sample size from one medical center. Some reported risk factors were not significant because the sample was relatively small such as insulin dependent DM [17]. For the same reason, we couldn’t reveal any risk factors of multiple complications, and the association between the application of iliac fixation and complications (*n* = 5 for multiple complications; *n* = 7 for application of iliac fixation). Otherwise, it was a retrospective cohort study, and there was lack of standardized comparison groups.

## Conclusion

The high morbidity of perioperative complications for posterior spinal fusion should draw more attention when planning operations in DS patients. It was independently associated with higher ASA grade and longer OD, respectively. For those high risk patients, such as ASA grade > 2, the optimized surgical strategies to shorten OD should be suggested to improve the patient outcomes.

## Abbreviations

95%CI: 95% Confidence Interval
Alb: Albumin
ASA: American Society of Anesthesiologists
BMI: Body Mass Index
CSF: Cerebrospinal Fluid
DM: Diabetes Mellitus
DS: Degenerative Scoliosis
Hgb: Hemoglobin
OD: Operation Duration
OR: Odds Ratio
RBC: Red Blood Cells
SRS: Scoliosis Research Society
